# Adiposity in relation to risks of fatty liver, cirrhosis and liver cancer: a prospective study of 0.5 million Chinese adults

**DOI:** 10.1038/s41598-018-36460-7

**Published:** 2019-01-28

**Authors:** Yuanjie Pang, Christiana Kartsonaki, Iain Turnbull, Yu Guo, Yiping Chen, Robert Clarke, Zheng Bian, Fiona Bragg, Iona Y. Millwood, Ling Yang, Ying Huang, Yan Yang, Xukui Zhang, Junshi Chen, Liming Li, Michael V. Holmes, Zhengming Chen

**Affiliations:** 1 Clinical Trial Service Unit & Epidemiological Studies Unit (CTSU), Nuffield Department of Population Health, University of Oxford, Oxford, UK; 2 Medical Research Council Population Health Research Unit (MRC PHRU), Nuffield Department of Population Health, University of Oxford, Oxford, UK; 3 Chinese Academy of Medical Sciences, 9 Dongdan San Tiao, Beijing, 100730 China; 4 Guangxi Zhuang Autonomous Region Center for Disease Control, Guangxi Province, Nanning, 530028 China; 5 Huixian People’s Hospital, Henan Province, Xinxiang, 453600 China; 6 Huixian Center for Disease Control, Henan Province Xinxiang, 453600 China; 7 National Center for Food Safety Risk Assessment, 37 Guangqu Road, Beijing, 100021 China; 8 School of Public Health, Peking University, Beijing, 100191 China; 9 National Institute for Health Research Oxford Biomedical Research Centre, Oxford University Hospital, Old Road, Oxford, OX3 7LE UK

## Abstract

Adiposity is an increasing public health problem in China. We aimed to examine the associations of adiposity with non-alcoholic fatty liver disease (NAFLD) and other chronic liver diseases in Chinese adults. The prospective China Kadoorie Biobank recruited 512,891 adults aged 30–79 years from 10 areas. During 10 years of follow-up, 7,386 incident liver disease cases were recorded among 503,991 participants without prior cancer or chronic liver disease at baseline. The mean body mass index (BMI) (SD) was 23.7 (3.3) kg/m^2^ and mean waist circumference (WC) 80.3 (9.8) cm, with 33% having BMI ≥25 kg/m^2^. Throughout the range examined (BMI 15–50) BMI showed a log-linear positive association with NAFLD (n = 1,298), with adjusted HR per 5 kg/m^2^ of 2.81 (95% CI 2.63–3.01), adjusting for regression dilution. There were also positive associations of percent body fat, WC, and waist-to-hip ratio with NAFLD, with HRs per 1-SD of 2.27 (2.14–2.41), 2.60 (2.44–2.76), and 1.84 (1.76–1.92). BMI was unrelated to viral hepatitis (n = 1,477), and had a U-shaped association with cirrhosis (n = 2,082) and an inverse association with liver cancer (n = 2,568), which disappeared after excluding the first 5 years of follow-up. Among Chinese adults, adiposity was a major risk factor for NAFLD but not other chronic liver diseases.

## Introduction

Worldwide about 50% of deaths from liver cancer and 15% from cirrhosis occurred in China, where chronic liver diseases, including chronic hepatitis B virus (HBV) infection, affect approximately 300 million people^1,2^. In recent decades, age-standardised adult mortality rates from liver cancer and cirrhosis have been declining in China, due partly to elimination of contamination of foodstuffs with aflatoxin. However, the incidence of non-alcoholic fatty liver disease (NAFLD) has been increasing^3^. Previous studies using ultrasound screening indicated that about 20% of Chinese adults may have NAFLD, with an annual incidence rate of 2–9%^3^. Individuals with NAFLD are also at higher risk of developing not only cirrhosis and liver cancer^4^, but also cardio-metabolic diseases^4^.

Worldwide the mean levels of adiposity have been increasing in recent decades^5^. Several prospective studies conducted in mainly East Asian populations have assessed the association of adiposity with risk of NAFLD^6–12^. Such studies have consistently demonstrated positive associations of body mass index (BMI) with risk of NAFLD. However, most of the previous studies were limited to occupational cohorts or high-risk individuals, and had relatively short duration of follow-up (usually <5 years) or a small number of NAFLD cases. Hence, substantial uncertainty persists about the shape and strength of the association of BMI with NAFLD, about the relative importance of different measures of adiposity (e.g. BMI versus waist circumference [WC]) for NAFLD^8,9,13^, and about the subsequent risks of cardio-metabolic disease, cirrhosis, and liver cancer following an initial diagnosis of NAFLD. Prospective studies in Western populations have shown a U-shaped association of BMI with risk of cirrhosis and a positive association with risk of liver cancer^14,15^. However, there is limited prospective evidence from East Asia, where aetiologies of liver diseases differ importantly from those in the West^16^.

In China, although the mean levels of BMI in adult populations are still much lower than in Western populations, a higher proportion tend to have central obesity^17^, which might predispose people to particularly high risk of NAFLD^18^. In Western populations, alcohol and NAFLD are the major risk factors for cirrhosis and liver cancer^16,19^, whereas in China HBV infection and exposure to aflatoxin are the most important risk factors^16,19^. Consequently, the association of adiposity with chronic liver diseases and prognosis of individuals with NAFLD may differ between Western and Chinese populations. Therefore, reliable prospective evidence about the relationship of adiposity with NAFLD and other liver diseases in China is needed to inform disease prevention strategies. We examined the associations of general (BMI, percent body fat [%BF], weight) and central adiposity (WC, hip circumference [HC], waist-to-hip ratio [WHR]) with NAFLD in the prospective China Kadoorie Biobank (CKB) study of 0.5 million adults. We also assessed the associations of BMI with other chronic liver diseases (cirrhosis, liver cancer, alcoholic liver disease [ALD], viral hepatitis, and other liver diseases).

## Results

Among the 503,991 participants included, the mean (SD) baseline age was 51.5 (10.7) years, and 59% were women. The mean (SD) measured BMI and WC at baseline were 23.7 (3.3) kg/m^2^ and 80.3 (9.8) cm, respectively. Overall, 33% were either overweight or obese (BMI ≥25 kg/m^2^). Participants with higher BMI were more likely to be female and from urban areas, and to have higher SBP and blood glucose. They were also more likely to have prevalent diabetes, a history of cardiovascular disease (CVD) and hypertension (Table 1).

**Table 1:** Baseline characteristics of study participants by BMI at baseline.

Variable^a^	BMI categories (kg/m^2^)						
	<20.0	20.0 to <22.5	22.5 to <25.0	25.0 to <27.5	27.5 to <30.0	≥30.0	All
	(n = 65,112)	(n = 129,561)	(n = 143,080)	(n = 99,889)	(n = 45,740)	(n = 20,609)	(n = 503,991)
Age (SD), year	52.4 (11.8)	50.7 (10.8)	51.0 (10.4)	51.5 (10.2)	51.6 (10.2)	51.6 (10.4)	**51**.**5** (**10**.**7**)
Female, %	56.0	57.2	59.7	59.9	62.7	71.0	**59**.**2**
**Socioeconomic and lifestyle factors**							
Urban resident, %	31.5	37.3	45.4	51.5	55.0	58.6	**44**.**1**
≥9 years of education, %	19.9	21.0	21.7	21.4	20.6	19.4	**20**.**9**
Household income ≥35 000 RMB/year, %	14.8	16.6	18.5	19.5	20.3	20.3	**17**.**9**
Ever regular smoking, %							
Male	76.1	71.5	65.8	62.6	61.6	61.6	**67**.**7**
Female	4.2	3.0	2.6	2.4	2.5	2.8	**2**.**8**
Weekly drinking, %							
Male	31.7	34.3	33.9	32.9	32.8	31.4	**33**.**5**
Female	2.1	2.1	2.1	2.0	2.1	1.7	**2**.**1**
Total physical activity (SD), MET h/day	21.4 (13.9)	21.8 (14.2)	21.3 (14.0)	20.6 (13.6)	19.9 (13.1)	19.0 (12.3)	**21**.**1** (**13**.**9**)
**Blood pressure**, **blood glucose and anthropometry**							
SBP (SD), mmHg	123.0 (20.9)	127.2 (20.4)	131.3 (20.6)	135.2 (20.8)	138.8 (21.1)	143.3 (21.9)	**131**.**1** (**21**.**3**)
RPG (SD), mmol/L	5.9	5.9	6.0	6.2	6.4	6.7	**6**.**1** (**2**.**3**)
Height (SD), cm	158.6 (8.3)	158.6 (8.1)	158.7 (8.2)	158.8 (8.4)	158.9 (8.5)	158.9 (8.5)	**158**.**7** (**8**.**3**)
BMI (SD), kg/m^2^	18.7	21.3	23.7	26.1	28.5	31.7	**23**.**7** (**3**.**4**)
Waist circumference (SD), cm	68.3 (5.2)	74.5 (5.3)	80.6 (5.8)	86.4 (6.0)	91.9 (6.3)	99.0 (7.6)	**80**.**3** (**9**.**8**)
Hip circumference (SD), cm	83.6 (4.3)	87.4 (4.2)	90.9 (4.4)	94.5 (4.6)	98.2 (4.8)	103.4 (6.3)	**90**.**9** (**6**.**9**)
Waist-to-hip ratio (SD)	0.82 (0.06)	0.85 (0.06)	0.89 (0.06)	0.92 (0.06)	0.94 (0.06)	0.96 (0.07)	**0**.**90** (**0**.**07**)
Percent body fat (SD), %	18.9 (4.9)	23.9 (5.5)	28.3 (5.8)	32.4 (6.3)	36.0 (6.9)	40.1 (7.9)	**28**.**0** (**8**.**4**)
BMI at age 25 (SD), kg/m^2^	20.5 (2.3)	21.4 (2.3)	21.9 (2.4)	22.5 (2.5)	23.1 (2.7)	23.9 (3.1)	**22**.**0** (**2**.**6**)
**HBsAg positive**, **%**	3.0	2.9	2.7	2.6	2.6	2.7	**2**.**7**
**Prior disease history**, **%**							
Diabetes	3.4	4.3	5.8	7.3	8.6	11.3	**5**.**9**
CHD	1.9	2.2	2.9	3.6	4.0	4.9	**5**.**9**
Stroke or TIA	1.0	1.4	1.8	2.0	2.2	2.7	**1**.**7**
Hypertension	4.6	7.3	11.2	16.0	20.4	27.2	**11**.**6**
Family history of diabetes	3.7	4.3	5.0	5.5	6.1	6.2	**4**.**9**
Family history of cancer	13.0	13.5	14.2	14.4	14.5	14.3	**13**.**9**

^**a**^Results were adjusted for age, region, and sex (where appropriate).

Abbreviations: MET = metabolic equivalent of task; SBP = systolic blood pressure; RPG = random plasma glucose; BMI = body mass index; HBsAg = hepatitis B surface antigen; CHD = coronary heart disease; TIA = transient ischaemic attack.

For weekly drinking, *p*-value for trend across BMI categories: 0.14 in men, 0.09 in women. For all other variables, *p*-value for trend across BMI categories: all <0.001.

**Figure 1:**
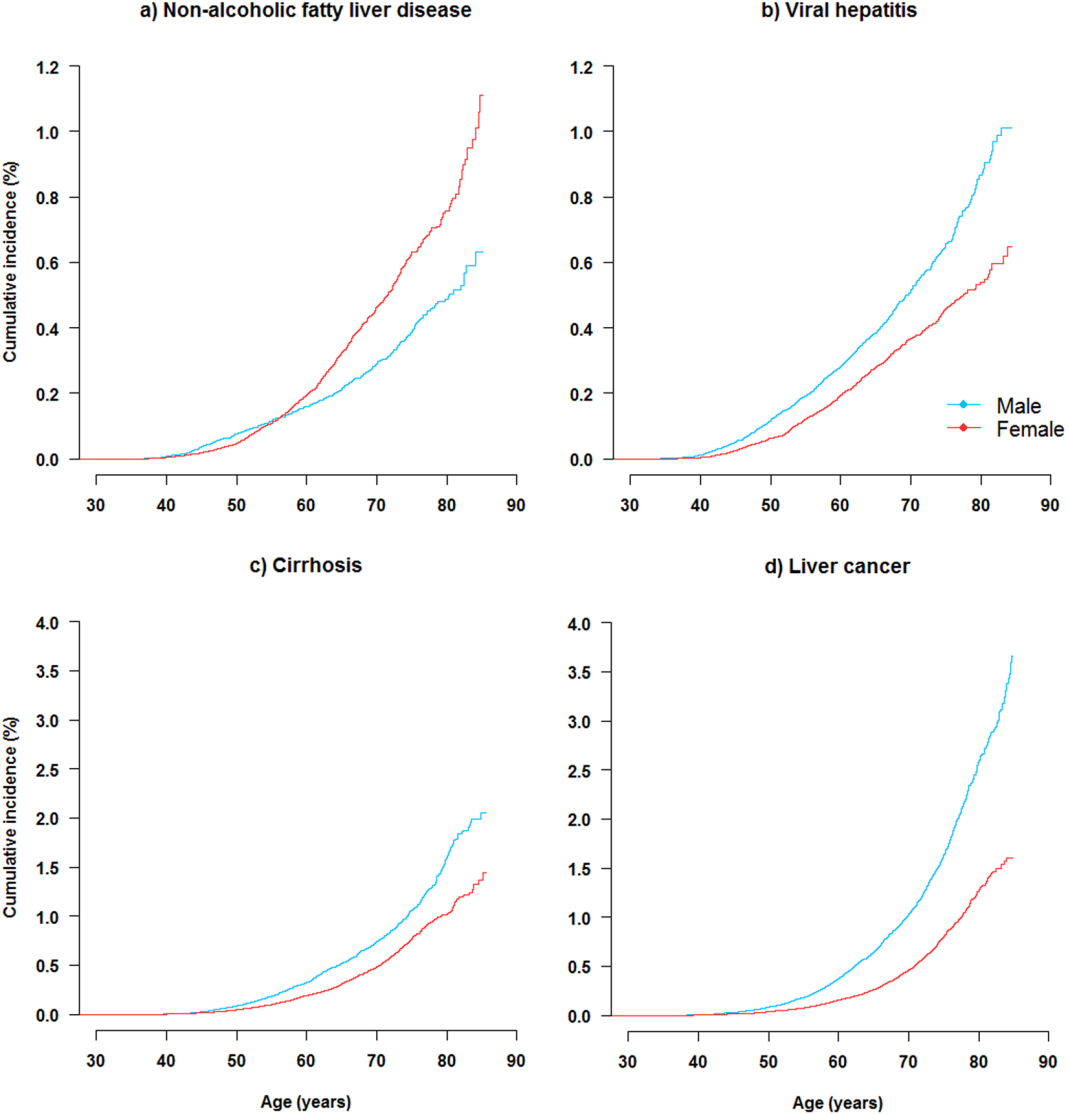
Cumulative incidence of NAFLD and other liver diseases among men and women. Survival curves to show event proportions of participants by age in males (blue) and females (red). Liver diseases include (**a**) NAFLD, (**b**) viral hepatitis, (**c**) cirrhosis, and (**d**) liver cancer. Curves are truncated at age 85.

During approximately 5.0 million person-years of follow-up, 7,386 participants developed liver diseases, including hospital-reported NAFLD (n = 1,298), cirrhosis (n = 2,082), hospital-reported ALD (n = 255), viral hepatitis (n = 1,477), and liver cancer (n = 2,568) at the age 30–89 years. The incidence was higher in males than in females for cirrhosis (Fig. 1), viral hepatitis, and liver cancer, while the incidence of NAFLD appeared to be higher in females after age ~60.

**Figure 2:**
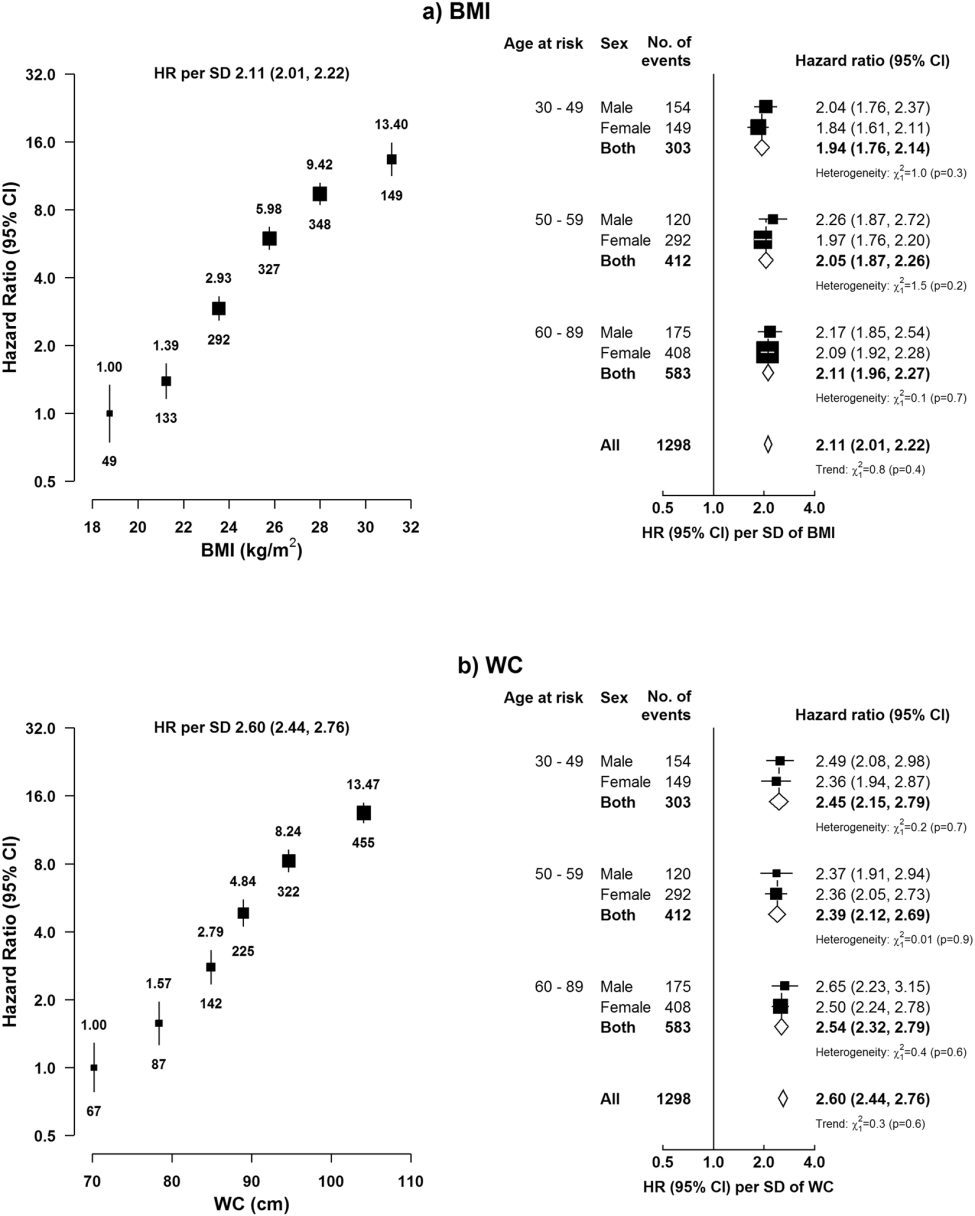
Adjusted HRs for hospital-reported NAFLD by usual level of (**a**) BMI and (**b**) WC, with the right panel showing the age and sex-specific HRs per 1-SD higher usual BMI and WC. In the left panel, BMI is classified as <20.0, 20.0 to <22.5, 22.5 to <25.0, 25.0 to <27.5, 27.5 to <30.0, and ≥30.0 kg/m^2^. WC is classified by sextiles as <70.7, 70.7–75.2 (reference), 75.3–79.6, 79.7–84.0, 84.1–89.9, and ≥90.0 cm. Hazard ratios (HRs) are plotted against the mean level in each group. Log-scale is used for the y-axis. The squares represent HRs, and the vertical lines represent 95% CIs. Numerical values above the 95% CI represent the hazard ratio and values beneath the 95% CI represent the number of cases of NAFLD in each group. In the right panel, boxes represent sex-specific estimates by age-at-risk categories. Diamonds represent summary HRs for each age-at-risk category and the overall HR. Estimates and 95% CI of the summary HRs are in bold. In both panels, the sizes of the boxes are proportional to the inverse of the variance of the log hazard ratios. The analyses are stratified by sex, region, and HBsAg, and adjusted for age at baseline, education, smoking, alcohol, and total physical activity. SD was 3.3 kg/m^2^ for BMI and 9.8 cm for WC. HR per 1-SD was corrected for regression dilution (regression dilution ratio: BMI = 0.93, WC = 0.84).

BMI showed a strong positive association with risk of hospital-reported NAFLD, with adjusted HRs of 2.78 (2.53–3.05), 9.99 (9.27–10.76), and 18.05 (15.34–21.23) for those with normal weight (i.e. 18.5–24.9 kg/m^2^), overweight (25–29.9 kg/m^2^), and obesity (≥30 kg/m^2^), compared with those who were underweight (i.e. BMI <18.5 kg/m^2^). Within the broad BMI range examined, the association with NAFLD was log-linear, with an adjusted HR of 2.11 (2.01–2.22) per 1-SD (3.3 kg/m^2^) higher usual BMI, corresponding to 2.81 (2.63–3.01) per 5 kg/m^2^ higher usual BMI (Table 2, Fig. 2).

**Table 2:** Standardised incidence rates and adjusted HRs for hospital-reported NAFLD and other liver diseases by baseline BMI^a^.

Liver disease	BMI categories (kg/m^2^)						P for trend
	<20.0	20.0 to <22.5	22.5 to <25.0	25.0 to <27.5	27.5 to <30.0	≥30.0	
	(n = 65,112)	(n = 129,561)	(n = 143,080)	(n = 99,889)	(n = 45,740)	(n = 20,609)	
**NAFLD**							
No. of cases	49	133	292	327	348	149	
Rate per 100,000	69.6	94.0	147.5	317.2	602.0	635.0	
HR (95% CI)	0.72 (0.54, 0.95)	1.00 (0.84, 1.19)	2.10 (1.87, 2.36)	4.34 (3.93, 4.79)	7.54 (6.71, 8.47)	9.64 (8.19, 11.33)	<0.001
**Cirrhosis**							
No. of cases	344	585	541	276	238	98	
Rate per 100,000	379.5	374.4	317.8	310.6	349.0	572.6	
HR (95% CI)^b^	1.02 (0.92, 1.14)	1.00 (0.92, 1.09)	0.91 (0.84, 0.99)	0.81 (0.72, 0.90)	0.99 (0.86, 1.15)	1.19 (0.97, 1.46)	0.42
**Liver cancer**							
No. of cases	417	700	693	380	280	98	
Rate per 100,000	583.1	563.6	509.1	459.2	476.7	464.0	
HR (95% CI)	1.02 (0.93, 1.13)	1.00 (0.93, 1.08)	0.96 (0.89, 1.03)	0.89 (0.81, 0.98)	0.92 (0.80, 1.05)	1.01 (0.82, 1.23)	0.02
**Viral hepatitis**							
No. of cases	198	363	425	253	176	62	
Rate per 100,000	262.0	239.2	264.8	267.5	226.3	308.4	
HR (95% CI)	1.06 (0.92, 1.22)	1.00 (0.90, 1.11)	1.12 (1.02, 1.23)	1.10 (0.98, 1.24)	1.16 (0.98, 1.38)	1.13 (0.87, 1.45)	0.50
**Other** ^**c**^							
No. of cases	544	1048	1012	634	436	142	
Rate per 100,000	677.1	702.6	596.9	673.1	672.1	750.7	
HR (95% CI)	0.96 (0.88, 1.05)	1.00 (0.94, 1.06)	0.92 (0.87, 0.98)	1.00 (0.93, 1.08)	1.03 (0.93, 1.15)	1.06 (0.90, 1.25)	0.33

Model was stratified by sex, region, and HBsAg, and adjusted for age at baseline, education, smoking, alcohol, and total physical activity. HR per 5 kg/m^2^ was corrected for regression dilution (regression dilution ratio = 0.93). Adjusted HRs for ALD were 1.50 (1.15–1.96), 1.00 (0.86–1.17), 0.72 (0.50–1.02), and 0.72 (0.45–1.16) for those with BMI of <20.0, 20.0 to <25.0, 25.0 to <27.5, and ≥27.5 kg/m^2^. Adjusted HR for ALD for 5 kg/m^2^ higher usual BMI was (0.59 [0.47–0.74], p<0.001).

^b^For cirrhosis, the HR per 5 units was 0.87 (0.77–0.99, p = 0.03) in the range 15–<25 kg/m^2^ and 1.37 (1.16–1.63, p < 0.001) in the range 25–50 kg/m^2^.

^c^Other liver diseases included ICD-10 code K72, K73, K75, K76.1–K76.5, and K76.7–K76.9.

**Figure 3:**
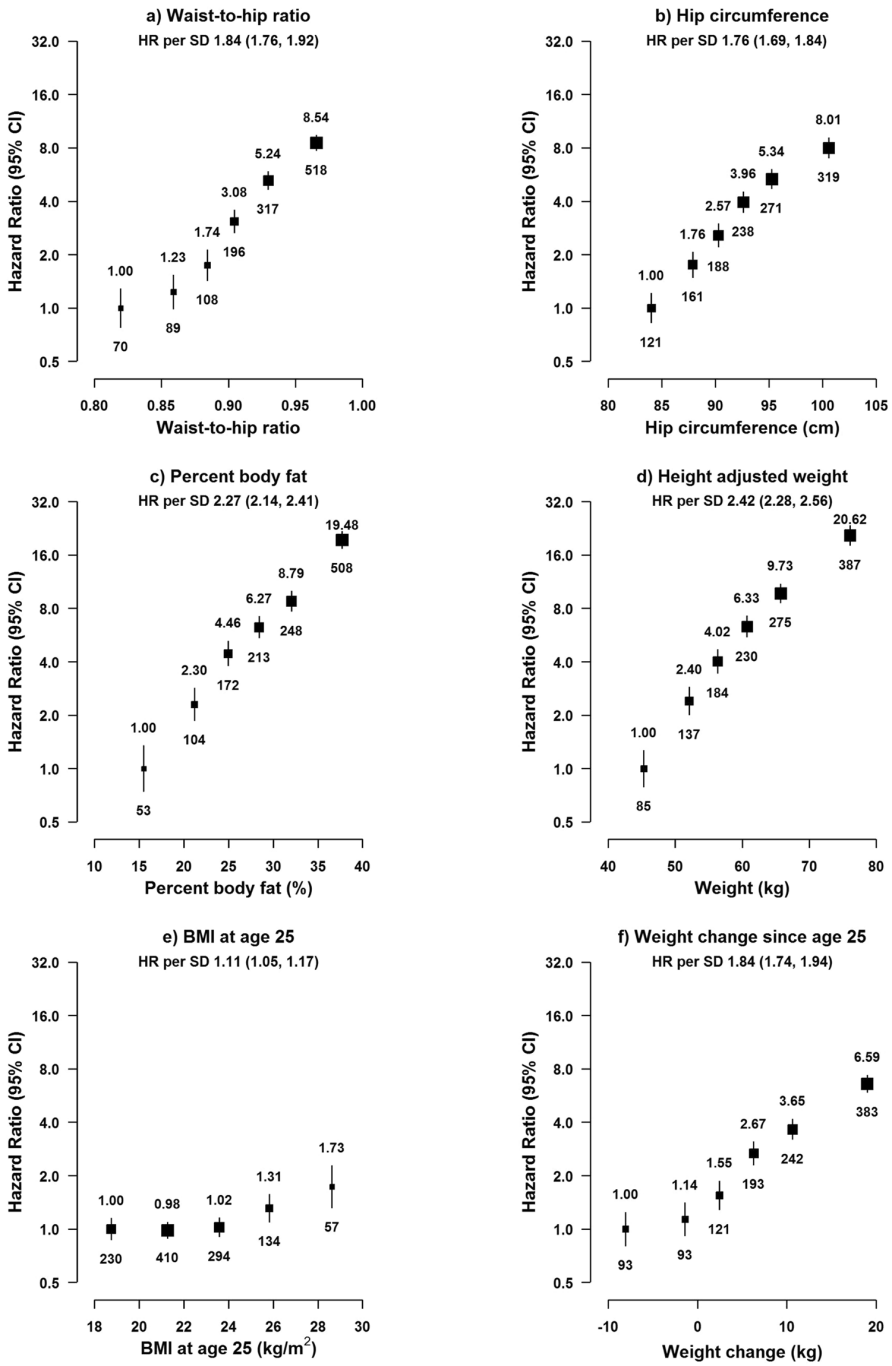
Adjusted HRs for NAFLD by usual level of other adiposity traits. Adiposity traits are modelled as sextiles and HRs are plotted against the mean level in each group. All adiposity traits are usual levels, except for BMI at age 25 and weight change since age 25. BMI at age 25 is missing in 81,409 participants. Log-scale is used for the y-axis. The squares represent HRs, and the vertical lines represent 95% CIs. The area of the squares is inversely proportional to the variance of the log HRs. Numerical values above the 95%CI represent the hazard ratio and values beneath the 95% CI represent the number of cases of NAFLD in each group. The analyses are stratified by sex, region, and HBsAg, and adjusted for age at baseline, education, smoking, alcohol, and physical activity. SD was 0.06 for WHR, 6.9 cm for HC, 8.4% for %BF, 10.8 kg for weight, 2.6 kg/m^2^ for BMI25, and 9.1 kg for weight change since age 25. HR per 1-SD was corrected for regression dilution (regression dilution ratio: WHR = 0.70, HC = 0.81, %BF = 0.88, weight = 0.96).

As with BMI, the risk of hospital-reported NAFLD increased progressively, again in a log-linear fashion, with other adiposity traits (Fig. 3, Supplementary Fig. 1). For WC and WHR, the adjusted HRs were 2.60 (2.44–2.76) and 1.84 (1.76–1.92) for each 1-SD increase in usual levels, respectively, while for %BF, the corresponding HR was 2.27 (2.14–2.41). When further adjusting for BMI, the positive associations remained for WC, WHR, and %BF (Supplementary Fig. 1). When adjusting for each other, the HR per 1-SD was greater for WC (2.04 [1.80–2.32]) than for BMI (1.26 [1.13–1.41]), consistent with the HRs per 1-SD without adjustment for each other. When simultaneously including BMI, %BF, WC, and HC in a model, the positive associations attenuated greatly for BMI and %BF (HR per 1-SD: BMI 1.28 [1.12–1.47], %BF 1.29 [1.15–1.44]) but only slightly for WC (HR per 1-SD: 2.11 [1.85–2.41]). When adjusting for BMI, %BF, and WC, the positive association for HC became inverse (HR per 1-SD: 0.77 [0.70–0.85]). For HC, the HR per 1-SD was 0.88 (0.80–0.97) when additionally adjusting only for WC. The associations of BMI and WC with hospital-reported NAFLD did not differ significantly by age, sex, and education (Supplementary Fig. 2), although the positive association appeared stronger in individuals from rural than urban areas and in individuals without a history of diabetes or CVD.

The association of BMI with cirrhosis appeared U-shaped, with an inverse association below 25 kg/m^2^ (HR per 5 kg/m^2^ 0.87 [0.77–0.99], *p* = 0.03) and a positive association above 25 kg/m^2^ (1.37 [1.16–1.63], p < 0.001; Table 2). There were inverse associations of BMI with risks of liver cancer (0.93 [0.87–0.99]) and hospital-reported ALD (0.59 [0.47–0.74]), but a null association with viral hepatitis (1.03 [0.95–1.11]). After excluding the first five years of follow-up (Supplementary Table S2), the association with cirrhosis was not substantially altered, but the inverse association with liver cancer disappeared completely. The associations of BMI with hospital-reported NAFLD and liver cancer were similar in men and in women (*p* for heterogeneity 0.34 and 0.91), whereas the association with cirrhosis appeared stronger in women (*p* for heterogeneity <0.001, Supplementary Table S3). In an analysis of 19,788 participants who attended the resurvey, there was a positive trend between BMI change with risk of hospital-reported NAFLD (HR per 5 kg/m^2^ 2.36 [0.77–7.22], 56 cases), but no significant associations with risks of cirrhosis (0.48 [0.16–1.43], 53 cases), liver cancer (0.61 [0.23–1.64], 73 cases), viral hepatitis (0.71 [0.21–2.33], 56 cases), and other liver diseases (0.87 [0.42–1.80], 130 cases), possibly due to the small numbers of cases. Smoking was associated with higher risk of liver cancer (1.34 [1.19–1.50]), while alcohol drinking was associated with higher risk of hospital-reported ALD (12.22 [7.18–20.82]) but not with other diseases (Supplementary Table S4). Individuals who tested positive for HBsAg at baseline had higher risks of cirrhosis (15.47 [13.97–17.14]), liver cancer (12.22 [11.11–13.47]), and viral hepatitis (24.54 [22.00–27.37]).

After diagnosis, individuals with hospital-reported NAFLD had a 2-fold higher risk of cardio-metabolic diseases compared to those without a NAFLD diagnosis (Supplementary Table S5). In addition, individuals with hospital-reported NAFLD had 3-fold higher risks of liver cancer and cirrhosis (Supplementary Table S5). Individuals with cirrhosis had higher risks of all-cause mortality and liver cancer, while individuals with viral hepatitis had higher risks of cirrhosis and liver cancer (Supplementary Table S5).

## Discussion

This is the first large prospective study of adiposity and risks of hospital-reported NAFLD and other chronic liver diseases in China. Among the relatively lean Chinese adults, there were strong positive log-linear associations of both general and central adiposity with an incident diagnosis of NAFLD, throughout the ranges studied for both BMI and WC. Individuals with a diagnosis of NAFLD had higher risks of developing cardio-metabolic diseases, as well as cirrhosis and liver cancer. In contrast to the strong positive association with hospital-reported NAFLD, BMI showed a U-shaped association with cirrhosis and an inverse association with liver cancer, which appeared to be driven chiefly by reverse causation. On the other hand, smoking, alcohol drinking, and HBV infection showed expected strong positive associations with certain chronic liver diseases in this population.

To date, seven prospective studies in East Asia involving ~16,000 NAFLD cases have shown reasonably consistently that a 1 kg/m^2^ higher BMI is associated with a 20–30% higher risk of ultrasound-detected NAFLD^6–12^. The results of the present study for hospital-reported NAFLD were consistent with those of previous studies using screen-detected NAFLD (1.23 vs 1.2–1.3 per 1 kg/m^2^ higher baseline BMI), but stronger than a UK study of hospital-reported NAFLD (1.23 [1.21–1.25] vs 1.15 [1.14–1.16] per 1 kg/m^2^ higher baseline BMI). Apart from BMI, three prospective studies have shown positive associations of WC and WHR with risk of ultrasound-detected NAFLD^8,9,13^, although there have been inconsistent findings regarding the relative predicting power of WC versus BMI^8,9^. Our study showed that WC was more strongly associated with NAFLD than BMI and that the association of WC persisted after adjustment for BMI, whereas the converse was not true for BMI after adjustment for WC, suggesting that WC may be a better predictor of NAFLD risk. In addition, we showed that %BF was most strongly associated with NAFLD among all adiposity measures considered, although the positive association was attenuated when further adjusting for BMI. In CKB, HC was positively associated with risk of NAFLD, and the positive association became inverse when additionally adjusting for WC. The reason for the inverse association of HC given WC is not well understood, but similar findings were also shown for diabetes in CKB^20^. It has been hypothesised that skeletal muscle mass is a positive regulator of systemic insulin sensitivity, and a large HC might indicate a great skeletal muscle mass in the gluteal region^21^.

Several large prospective studies conducted in North America and Europe have shown a U-shaped association of BMI with risk of cirrhosis^14,15,22^, while the only East Asian study (314 deaths from hepatitis or cirrhosis [ICD-10 K70-K77]) has shown no association^23^. Previous studies in Western populations included ALD (K70), accounting for 40–60% of chronic liver diseases in North America or Europe^19^. In China, HBV is the major cause of cirrhosis, with ALD accounting for a much smaller proportion of chronic liver diseases (4% in CKB [7% in men])^1^. Despite the different aetiology of cirrhosis, our findings were consistent with previous studies in Western populations. The UK Million Women Study (MWS, 1.2 M participants and 1,811 cases)^14^ and the Prospective Study Collaboration (PSC, 0.9 M participants and 914 cases)^15^ reported an inverse association between BMI and cirrhosis at low BMI levels (15 to <25 kg/m^2^) and a positive association at high levels (25–50 kg/m^2^), and both trends remained after excluding early years of follow-up. Unlike CKB, PSC used mortality rather than incidence, and when cases from the first five years were excluded, there were stronger associations at both low and high BMI levels (HR per 5 kg/m^2^ 15–25 0.73 vs 0.91, 25–50 1.79 vs 1.45). Although similar approaches were used to identify incident cirrhosis cases in MWS (i.e. hospital admissions and death certificates), we showed a stronger association between BMI and cirrhosis risk than MWS (HR per 5 kg/m^2^ ~25–50 1.28 vs 1.37 in CKB). This is probably because we used measured BMI and corrected for regression dilution, while MWS used self-reported BMI. In CKB, we assessed ALD as a separate outcome, and observed an inverse association between BMI and ALD. Our findings differed from two small cross-sectional studies in China that reported a positive association of BMI with alcoholic fatty liver disease (AFLD)^24,25^. However, the majority of ALD cases in CKB had cirrhosis (~70%), and therefore it is possible that the associations of BMI with AFLD and alcoholic cirrhosis differed. Moreover, given the small number of cases involved, we cannot completely exclude the possibility that the inverse association in CKB may be a chance finding.

Previous prospective studies conducted in Western populations have shown that BMI is positively associated with risk of liver cancer. However, the majority of prospective studies conducted in East Asia have reported no association^26^, while some studies reported higher risks in both underweight (BMI <20 kg/m^2^) and obese (BMI ≥30 kg/m^2^) participants^26^. In contrast to CKB, six out of 11 East Asian studies did not exclude early years of follow-up^26^, and therefore the higher risk for participants with low BMI may reflect reverse causality. Liver cancer has a long latency period, and it is possible that individuals with undetected cancer at study baseline may lose weight, thereby resulting in a false negative association of BMI with liver cancer. In CKB, the inverse association of BMI and liver cancer risk was attenuated and became non-significant when the first five years of follow-up were excluded. Furthermore, it has been hypothesised that obesity is linked to liver cancer through development of NAFLD and progression to cirrhosis^26^. In Western populations, obesity plays an important role in the aetiology of liver cancer where NAFLD is the main cause of liver cancer^16^. The situation is different in China and many other East Asian populations, where up to 40% of cases with HBV-related liver cancer do not have underlying cirrhosis^16^. The discrepant causes of liver cancer may explain the different associations of BMI with liver cancer in East Asia and North America or Europe. Nonetheless, despite the small proportion of liver cancer cases attributable to NAFLD in the Chinese population, we observed that NAFLD cases had a higher risk developing liver cancer.

Apart from the prospective study design and large sample size, the main strength of the present study was linkage to hospital records in addition to death and cancer registries, permitting exploration of the underlying causes of liver diseases. In addition, our study assessed a range of adiposity measures, along with other risk factors (e.g. smoking, alcohol, and HBsAg) with hospital-reported NAFLD and other liver diseases simultaneously. However, there were several limitations. First, rather than screening for NAFLD, our study relied on hospital records to capture NAFLD cases, which may have resulted in under-diagnosis. Previous prospective studies in China that screened for NAFLD using ultrasound reported an annual incidence of 2–9%^3^, while in CKB the incidence of hospital-reported NAFLD was 0.03%. However, we ascertained all NAFLD cases diagnosed between 2013 and 2015 and showed that 93% of all NAFLD cases were diagnosed by ultrasound or CT. Moreover, the agreement of our risk estimates with previous studies of ultrasound-detected NAFLD and the consistency of our risk estimates across population subgroups (Supplementary Fig. 2) suggest that our results for NAFLD are probably real. Second, the use of self-report may have underestimated prevalent liver diseases at baseline. However, in a nested case-control study of ~18,000 participants with blood biochemistry data, we showed similar associations between adiposity and NAFLD when excluding participants above the top quintile of liver enzymes (Supplementary Table S6). Third, cases hospitalised for liver disease or cardio-metabolic disease may be more likely to be screened for presence of NAFLD, potentially introducing detection bias. However, we found similar associations between adiposity and incident diagnosis of NAFLD when censoring cases with comorbidities prior to NAFLD diagnosis (Supplementary Table S7).

In conclusion, among relatively lean Chinese adults, adiposity, irrespective of how it was measured, was associated strongly and positively with hospital-reported NAFLD. However, BMI was not associated with liver cancer or other chronic liver diseases. On the other hand, smoking, alcohol drinking, and HBV infection were not associated with hospital-reported NAFLD, but showed expected associations with several other liver diseases. Overall, our findings suggest that a lower BMI, even within the so-called normal range, should confer a lower risk of NAFLD. Public health efforts to curb obesity are needed to prevent NAFLD, as well as its long-term adverse risks of diabetes, CVD, cirrhosis, and liver cancer.

## Methods

### Study population

Details of the CKB design, survey methods, and population characteristics have been described elsewhere^18^. Briefly, 512,891 participants (210,222 men and 302,669 women) aged 30–79 were recruited into the study from 10 (5 urban, 5 rural) localities in China during 2004–2008. The study areas were selected to provide diversity in risk factor exposure and disease patterns, while taking into account population stability, quality of mortality and morbidity registries, capacity, and long-term commitment within the areas. The CKB study was approved by the Ethical Review Committee of the Chinese Centre for Disease Control and Prevention (CDC) and the Oxford Tropical Research Ethics Committee, University of Oxford. All participants eligible for this study had completed a written informed consent form. All methods were performed in accordance with relevant guidelines and regulations.

At local study assessment clinics, participants completed an interviewer-administered laptop-based questionnaire on socio-demographic characteristics, smoking, alcohol consumption, diet, physical activity, personal and family medical history and current medication. A range of physical measurements were recorded by trained technicians, including height, weight, HC, WC, bio-impedance, lung function, blood pressure and heart rate, using calibrated instruments with standard protocols. In addition, desktop analysers were used to measure random blood glucose and hepatitis B surface antigen (HBsAg) (ACON Biotech).

All anthropometric measurements were taken by trained technicians while participants were wearing light clothes and no shoes, usually to the nearest 0.1 cm or 0.1 kg. Standing height was measured using a stadiometer. Weight was measured using a body composition analyser (TANITA-TBF-300GS; Tanita Corporation), with subtraction of weight of clothing according to season (ranging from 0.5 kg in summer to 2.0–2.5 kg in winter). WC and HC were measured using a soft non-stretchable tape, with HC measured at the maximum circumference around the buttocks. Adulthood BMI was calculated as the measured weight in kilograms divided by the square of the measured height in metres. BMI at age 25 (BMI25) was calculated using the recalled weight at age 25 years and the measured height at baseline. BMI change was calculated as the difference between BMI at baseline and BMI25 (i.e. BMI25 subtracted from BMI at baseline). WHR was the ratio of WC to HC. %BF was the fraction of total weight that was estimated to be fat weight by the Tanita body composition analyser using proprietary algorithms.

From August to October 2008 (~2.6 years after the baseline survey) 19,788 (~5%) surviving participants were randomly selected to attend a resurvey. The data collection and survey procedures were much the same as in the baseline survey. Adiposity measurements were available for 19,788 (100%) resurvey participants.

### Follow-up for mortality and morbidity

The vital status of each participant was determined periodically through China CDC’s Disease Surveillance Points (DSP) system^19^, supplemented by regular checks against local residential records and health insurance records and by annual active confirmation through street committees or village administrators. Additional information about major diseases and any episodes of hospitalisation was collected through linkages, via each participant’s unique national identification number, with disease registries (for cancer, IHD, stroke, and diabetes) and national health insurance claims databases (for liver diseases), which has almost universal coverage in the study areas. All events were coded using International Classification of Diseases, 10th Revision (ICD-10) by trained staff who were blinded to baseline information^18^. The classifications of liver diseases and distributions of outcome ascertainment by sources are shown in Supplementary Table S1 in the Supplement (NAFLD and ALD 100% by medical records). By 1.1.2016, 37,289 (7%) participants had died and 4,875 (<1%) were lost to follow-up.

### Statistical analysis

The present study excluded individuals with a prior history of cancer (n = 2,577), cirrhosis or hepatitis at baseline (n = 6,321), and missing data on BMI (n = 2). After these exclusions, 503,991 individuals remained in the main analyses.

The mean values and prevalence of baseline characteristics were calculated by adulthood BMI categories, standardised by 5-year age groups, sex, and study area. Incidence rates of chronic liver diseases were calculated using direct standardisation to the age, sex, and area structure of the population, where appropriate.

Cox regression models were used to estimate adjusted hazard ratios (HRs) of liver disease associated with adiposity, stratified by sex, region, and HBsAg, and adjusted for age at baseline, education, smoking, alcohol, and total physical activity. Time since birth was used as the underlying time scale with delayed entry at age at baseline. Cox models with a time-updated exposure were used to estimate HRs between incident NAFLD and subsequent risk of death and incidence of cardio-metabolic disease or cirrhosis and liver cancer, with the same adjustment. Incident NAFLD status was a time-dependent variable and individuals were considered as exposed from the time of diagnosis. BMI was modelled as a categorical variable with six categories (<20.0, 20.0 to <22.5, 22.5 to <25.0, 25.0 to <27.5, 27.5 to <30.0, and ≥30.0 kg/m^2^). BMI was also modelled as a continuous variable to estimate effects per 5 kg/m^2^ or per one standard deviation (SD) increase. For height-adjusted weight, standing height was also included in the model as a continuous variable. Other measures of adiposity were modelled by splitting at sextiles and as continuous. To further examine the associations of adiposity traits with risk of NAFLD, BMI, WC, HC, and %BF were simultaneously included in the model as well as other covariates (i.e. age at baseline, education, smoking, and alcohol) and strata (sex, region, and HBsAg). HRs per 1-SD increment of each adiposity trait were reported. To examine the associations of other possible risk factors for NAFLD, smoking, alcohol, and HBsAg were simultaneously included in the model as well as other covariates. HRs for each anthropometric category are presented along with the variance of the log risk in each category using so-called ‘floating’ standard errors, so that each HR has a 95% confidence interval (CI) that facilitates comparisons between any two groups^20^.

To adjust for regression dilution bias and obtain the association between usual levels of adiposity-related traits and risk of liver diseases, we calculated the correlation between all adiposity measures at baseline and first resurvey among 19,788 participants. Log HR estimates (and corresponding standard errors) per 1 SD higher adiposity measures (and per 5 kg/m^2^ for BMI) were divided by this correlation to obtain regression dilution-adjusted estimates^21^. Statistical analysis was done using SAS version 9.3 and R version 2.14.2.

## Electronic supplementary material

Supplementary materials

## Data Availability

The datasets analyzed during the current study are available from the corresponding author on reasonable request.
