# Supplementary material for: Adiposity in relation to risks of fatty liver, cirrhosis and liver cancer: a prospective study of 0.5 million Chinese adults
Source: Sci Rep. 2019 Jan 28;9:785. doi: 10.1038/s41598-018-36460-7 (PMC6349919; doi:10.1038/s41598-018-36460-7)
Supplement: Supplementary file 1 — Supplementary materials [file 41598_2018_36460_MOESM1_ESM.docx]

**Supplementary Materials**

**Adiposity in relation to risks of fatty liver, cirrhosis and liver cancer:**

**a prospective study of 0.5 million Chinese adults**

**Scientific Reports**

Yuanjie Pang^1^, ScM; Christiana Kartsonaki^1,2^, DPhil; Iain Turnbull^1^, MRCP; Yu Guo^3^, MSc;

Yiping Chen^1,2^, DPhil; Robert Clarke^1^, FRCP; Zheng Bian^3^, MSc; Fiona Bragg^1^, DPhil;

Iona Y Millwood^1,2^, DPhil; Ling Yang^1,2^, PhD; Ying Huang^4^, MSc; Yan Yang^5^, BSc; Yukui Zhang^6^, BSc; Junshi Chen^7^, MD; Liming Li^3,8^, MD; Michael V Holmes^1,2,9^, PhD; Zhengming Chen^1^, DPhil

1. Clinical Trial Service Unit & Epidemiological Studies Unit (CTSU), Nuffield Department of Population Health, University of Oxford, Oxford, UK
2. Medical Research Council Population Health Research Unit (MRC PHRU), Nuffield Department of Population Health, University of Oxford, Oxford, UK
3. Chinese Academy of Medical Sciences, 9 Dongdan San Tiao, Beijing 100730, China
4. Guangxi Zhuang Autonomous Region Center for Disease Control, Guangxi Province 530028, China
5. Huixian People’s Hospital, Henan Province 453600, China
6. Huixian Center for Disease Control, Henan Province 453600, China
7. National Center for Food Safety Risk Assessment, 37 Guangqu Road, Beijing 100021, China
8. School of Public Health, Peking University, Beijing 100191, China
9. National Institute for Health Research Oxford Biomedical Research Centre, Oxford University Hospital, Old Road, Oxford OX3 7LE, UK

**Address for correspondence**

Dr Christiana Kartsonaki

CTSU, Big Data Institute Building

Old Road Campus

University of Oxford

Oxford, OX3 7LF, UK

Fax: 44-1865-743985

Email: christiana.kartsonaki@ndph.ox.ac.uk

**Table of Contents**

[eMethods 3](#_Toc529541205)

[Supplementary Table S1. Classification and distribution of liver diseases by ICD-10 code in CKB 5](#_Toc529541206)

[Supplementary Table S2. Adjusted HRs for cirrhosis and liver cancer by baseline BMI after excluding the first 5 years of follow-up 6](#_Toc529541207)

[Supplementary Table S3. Standardised incidence rates and adjusted HRs for NAFLD and other liver diseases by baseline BMI 7](#_Toc529541208)

[Supplementary Table S4. Adjusted HRs for liver diseases associated with smoking, alcohol drinking and HBV infection in CKB 8](#_Toc529541209)

[Supplementary Table S5. Adjusted HRs for deaths and major disease outcomes following hospital diagnosis of NAFLD, cirrhosis, and viral hepatitis 9](#_Toc529541210)

[Supplementary Table S6. Adjusted HRs for NAFLD by baseline adiposity excluding participants in the top quintile of liver enzymes 10](#_Toc529541211)

[Supplementary Table S7. Adjusted HRs for NAFLD by adiposity when censoring cases with comorbidities prior to NAFLD diagnosis 11](#_Toc529541212)

[Supplementary Figure S1. Adjusted HRs for NAFLD associated with 1-SD increase in usual level of different measures of adiposity 12](#_Toc529541213)

[Supplementary Figure S2. Adjusted HRs for NAFLD associated with 1-SD increase in usual levels of a) BMI and b) WC in selected subgroups of study population 13](#_Toc529541214)

# eMethods

*Nested case-control study*

The nested case-control study involved stroke and coronary heart disease (CHD) subtypes among 18,183 CKB individuals, and included genome-wide array data and standard clinical biochemistry analysis. Cases were identified as those that had an incident fatal or nonfatal event coded as ICD-10: I21-23 for myocardial infarction (MI, n=1273); I63 and I69.3 for ischemic stroke (IS, n=5447); I61 for intracerebral hemorrhage (ICH, n=5150) at the censoring date of 1 January 2015. Cases were selected from the larger study as those with the youngest age at event. Common controls (n=6313) were frequency matched to cases by age, sex and area. Cases and controls were free of prior vascular disease (including absence of statin therapy) and cancer.

*Measurement of NMR-metabolomics and clinical chemistry*

The stored baseline plasma samples were retrieved, thawed and sub-aliquoted at the Wolfson laboratory, CTSU, and 100 uL aliquots were shipped on dry ice to the Brainshake Laboratory at Oulu, Finland, for NMR spectroscopy. Eight traits (total cholesterol, LDL-C, HDL-C, TGs, apolipoprotein B, apolipoprotein-A1, albumin and creatinine) were quantified using standard clinical biochemistry assays at the Wolfson Laboratory, CTSU, University of Oxford, UK. Plasma concentrations of total cholesterol, TGs, apolipoproteins B and A1 in EDTA plasma were measured by Beckman-Coulter AU680 clinical chemistry analysers using manufacturers’ reagents, calibrators and settings (Beckman-Coulter, UK), except LDL-C and HDL-C which used N-geneous reagents, calibrators and settings (Genzyme Diagnostics, UK).

Reference:

MV Holmes, IY Millwood, C Kartsonaki, MR Hill, DA Bennett, R Boxall, X Xu, B Zheng, R Hu, RG Walters, J Chen, M Ala-Korpela, S Parish, RJ Clarke, R Peto, R Collins, L Li, Z Chen. Lipoprotein particles, lipids and other metabolites and risks of incident myocardial infarction and stroke subtypes. *J Am Coll Cardiol*. 2018; 71(6): 620-32.

# Supplementary Table S1. Classification and distribution of liver diseases by ICD-10 code in CKB

| **Liver disease** | **ICD-10 code** | **No. of cases** | | | |
| --- | --- | --- | --- | --- | --- |
|  |  | **Total** | **Health insurance** | **Death registry** | **Disease registry** |
| NAFLD | K76.0 | 1298 | 1298 | 0 | 0 |
| Cirrhosis | K74 | 2082 | 1603 | 713 | 5 |
| Liver cancer | C22 | 2568 | 1178 | 1847 | 974 |
| Alcoholic liver diseases | K70 | 255 | 255 | 0 | 0 |
| Viral hepatitis | B18, B19 | 1477 | 1118 | 382 | 1 |
| Other liver diseases | K72, K73, K75, K76.1-K76.9 | 3816 | 3623 | 221 | 3 |

# Supplementary Table S2. Adjusted HRs for cirrhosis and liver cancer by baseline BMI after excluding the first 5 years of follow-up^a^

|  |  |  | **BMI** | |  |  | **P for** |
| --- | --- | --- | --- | --- | --- | --- | --- |
|  | **<20.0** | **20.0 to <22.5** | **22.5 to <25.0** | **25.0 to <27.5** | **27.5 to <30.0** | **≥30.0** |  |
|  | (n=64,469) | (n=129,306) | (n=142,806) | (n=99,674) | (n=45,659) | (n=20,571) | **Trend** |
| **Cirrhosis** |  |  |  |  |  |  |  |
| No. of cases | 191 | 307 | 328 | 161 | 153 | 59 |  |
| Rate per 100,000 | 473.0 | 410.2 | 377.5 | 355.6 | 490.2 | 615.7 |  |
| HR (95% CI)^b^ | 1.13 (0.98, 1.31) | 1.00 (0.89, 1.12) | 1.04 (0.93, 1.16) | 0.88 (0.76, 1.02) | 1.23 (1.03, 1.47) | 1.33 (1.03, 1.73) | 0.58 |
|  |  |  |  |  |  |  |  |
| **Liver cancer** |  |  |  |  |  |  |  |
| No. of cases | 182 | 381 | 388 | 220 | 158 | 62 |  |
| Rate per 100,000 | 502.8 | 607.5 | 546.7 | 494.9 | 575.9 | 552.5 |  |
| HR (95% CI) | 0.85 (0.73, 0.98) | 1.00 (0.90, 1.11) | 0.97 (0.88, 1.07) | 0.90 (0.80, 1.02) | 0.99 (0.83, 1.18) | 1.16 (0.90, 1.50) | 0.58 |

**^a^** Model was stratified by age-at-risk, sex, region, and HBsAg, and adjusted for age at baseline, education, smoking, alcohol, and total physical activity. HR per 5 kg/m^2^ was corrected for regression dilution (regression dilution ratio=0.935).

^b^ For cirrhosis, the HR per 5 units was 0.92 (0.78-1.09, p=0.35) in the range 15-<25 kg/m^2^ and 1.40 (1.13-1.74, p=0.002) in the range 25-50 kg/m^2^.

# Supplementary Table S3. Standardised incidence rates and adjusted HRs for NAFLD and other liver diseases by baseline BMI^a^

|  |  |  | **BMI categories** | |  |  | ***p* for** |
| --- | --- | --- | --- | --- | --- | --- | --- |
|  | **<20.0** | **20.0 to <22.5** | **22.5 to <25.0** | **25.0 to <27.5** | **27.5 to <30.0** | **≥30.0** |  |
|  | (n=64,712) | (n=129,570) | (n=143,053) | (n=99,886) | (n=45,730) | (n=20,484) | **trend** |
| **NAFLD** |  |  |  |  |  |  |  |
| **Men** |  |  |  |  |  |  |  |
| No. of cases | 19 | 55 | 90 | 127 | 118 | 40 |  |
| Rate per 100,000 | 53.0 | 102.2 | 133.6 | 336.2 | 577.1 | 634.5 |  |
| HR (95% CI) | 0.70 (0.44, 1.10) | 1.00 (0.76, 1.31) | 1.66 (1.35, 2.04) | 4.11 (3.50, 4.82) | 6.44 (5.26, 7.88) | 8.12 (5.93, 11.13) | <0.001 |
| **Women** |  |  |  |  |  |  |  |
| No. of cases | 30 | 78 | 202 | 200 | 230 | 109 |  |
| Rate per 100,000 | 80.9 | 88.4 | 157.0 | 304.4 | 619.0 | 635.5 |  |
| HR (95% CI) | 0.73 (0.51, 1.04) | 1.00 (0.80, 1.25) | 2.36 (2.06, 2.72) | 4.36 (3.84, 4.95) | 7.91 (6.86, 9.12) | 10.07 (8.33, 12.18) | <0.001 |
|  |  |  |  |  |  |  |  |
| **Cirrhosis** |  |  |  |  |  |  |  |
| **Men** |  |  |  |  |  |  |  |
| No. of cases | 211 | 362 | 276 | 141 | 110 | 31 |  |
| Rate per 100,000 | 529.6 | 543.9 | 414.4 | 396.1 | 397.7 | 458.9 |  |
| HR (95% CI) | 1.01 (0.88, 1.16) | 1.00 (0.90, 1.11) | 0.81 (0.72, 0.91) | 0.74 (0.63, 0.86) | 0.82 (0.66, 1.03) | 0.91 (0.64, 1.30) | <0.001 |
| **Women** |  |  |  |  |  |  |  |
| No. of cases | 133 | 223 | 265 | 135 | 128 | 67 |  |
| Rate per 100,000 | 277.7 | 259.4 | 252.2 | 252.5 | 316.0 | 436.4 |  |
| HR (95% CI)^2^ | 1.05 (0.88, 1.25) | 1.00 (0.88, 1.14) | 1.05 (0.93, 1.18) | 0.90 (0.77, 1.05) | 1.20 (0.99, 1.45) | 1.46 (1.14, 1.87) | 0.07 |
|  |  |  |  |  |  |  |  |
| **Liver cancer** |  |  |  |  |  |  |  |
| **Men** |  |  |  |  |  |  |  |
| No. of cases | 259 | 489 | 434 | 236 | 157 | 45 |  |
| Rate per 100,000 | 879.5 | 927.3 | 806.2 | 734.6 | 704.8 | 617.6 |  |
| HR (95% CI) | 0.91 (0.80, 1.04) | 1.00 (0.91, 1.09) | 0.91 (0.83, 1.00) | 0.86 (0.76, 0.97) | 0.83 (0.68, 0.99) | 0.94 (0.70, 1.26) | 0.05 |
| **Women** |  |  |  |  |  |  |  |
| No. of cases | 158 | 211 | 259 | 144 | 123 | 53 |  |
| Rate per 100,000 | 383.2 | 316.8 | 307.4 | 272.2 | 321.9 | 359.7 |  |
| HR (95% CI) | 1.28 (1.09, 1.50) | 1.00 (0.87, 1.15) | 1.05 (0.93, 1.19) | 0.97 (0.83, 1.12) | 1.09 (0.89, 1.33) | 1.14 (0.87, 1.50) | 0.13 |

**^a^** Model was stratified by age-at-risk, sex, region, and HBsAg, and adjusted for age at baseline, education, smoking, alcohol, and total physical activity. HR per 5 kg/m^2^ was corrected for regression dilution (regression dilution ratio=0.93).

*p* for heterogeneity by sex: NAFLD, 0.34; cirrhosis, 0.0003, liver cancer, 0.91.

# Supplementary Table S4. Adjusted HRs for liver diseases associated with smoking, alcohol drinking and HBV infection^a^

|  | **No. of**  **cases** | **Current vs never smokers** | **Weekly vs non-drinkers** | **HBsAg seropositivity** |
| --- | --- | --- | --- | --- |
|  |  | **HR (95% CI)** | **HR (95% CI)** | **HR (95% CI)** |
| NAFLD | 1298 | 1.39 (1.17, 1.65) | 1.13 (0.94, 1.37) | -- |
| Cirrhosis | 2082 | 1.21 (1.06, 1.38) | 1.02 (0.88, 1.18) | 15.47 (13.97, 17.14) |
| Liver cancer | 2568 | 1.34 (1.19, 1.50) | 1.03 (0.91, 1.17) | 12.22 (11.11, 13.47) |
| Viral hepatitis | 1477 | 1.17 (0.99, 1.38) | 0.89 (0.74, 1.07) | 24.54 (22.00, 27.37) |
| ALD | 255 | 1.50 (0.99, 2.24) | 12.22 (7.18, 20.82) | -- |
| Other | 3816 | 0.96 (0.87, 1.06) | 0.94 (0.84, 1.04) | 6.79 (6.18, 7.46) |

**^a^** Model was stratified by age-at-risk, sex, region, and HBsAg, and adjusted for age at baseline, education, smoking, alcohol, and total physical activity, where appropriate.

Analyses for smoking and alcohol were restricted to male participants only.

# Supplementary Table S5. Adjusted HRs for deaths and major disease outcomes following hospital diagnosis of NAFLD, cirrhosis, and viral hepatitis^a^

| **Outcome** | **NAFLD** | |  | **Cirrhosis** | |  | **Viral hepatitis** | |
| --- | --- | --- | --- | --- | --- | --- | --- | --- |
|  | **No. cases**^b^ | **HR (95% CI)** |  | **No. cases** | **HR (95% CI)** |  | **No. cases** | **HR (95% CI)** |
| CVD^c^ | 176/1122 | 1.74 (1.48, 2.05) |  | 245/1837 | 1.73 (1.49, 2.00) |  | 152/1325 | 1.52 (1.23, 1.89) |
| Diabetes^d^ | 123/1175 | 2.87 (2.35, 3.50) |  | 59/2023 | 1.48 (1.10, 2.00) |  | 39/1441 | 0.98 (0.62, 1.57) |
| Cardio-metabolic^e^ | 243/1055 | 2.27 (1.97, 2.61) |  | 333/1749 | 1.56 (1.30, 1.87) |  | 189/1258 | 1.52 (1.24, 1.85) |
| Cirrhosis | 8/1290 | 3.77 (1.88, 7.57) |  | **—** | **—** |  | 123/1354 | 11.10 (8.21, 15.23) |
| Liver cancer | 7/1291 | 2.67 (1.20, 5.97) |  | 197/1885 | 12.40 (9.35, 16.44) |  | 67/1358 | 3.08 (1.97, 4.82) |
| Any death | 66/1232 | 1.31 (1.00, 1.71) |  | 253/1829 | 4.95 (4.24, 5.78) |  | 154/1323 | 1.87 (1.41, 2.49) |

**^a^** Model was stratified by age-at-risk, sex, region, and HBsAg, and adjusted for age at baseline, education, smoking, alcohol, and total physical activity.

^b^ Number of NAFLD cases that had or had not developed major disease outcomes.

^c^ CVD: non-fatal and fatal events (I00-I09, I16-I25, I27-I88, I95-I99), fatal events (I10-I15).

^d^ Diabetes: non-fatal and fatal events (E10-E14).

^e^ Cardio-metabolic: CVD and diabetes.

# Supplementary Table S6. Adjusted HRs for NAFLD by baseline adiposity excluding participants in the top quintile of liver enzymes^a^

|  | **Nested case-control** | **Exclusion by ALT^b^** | **Exclusion by AST^c^** | **Exclusion by GGT^d^** |
| --- | --- | --- | --- | --- |
|  | **HR (95% CI)** | **HR (95% CI)** | **HR (95% CI)** | **HR (95% CI)** |
| **No. of participants** | 17 972 | 12 137 | 12 251 | 11 781 |
| **No. of cases** | 51 | 23 | 26 | 24 |
| **BMI per 1-SD** | 2.03 (1.56, 2.63) | 2.02 (1.34, 3.03) | 1.87 (1.28, 2.73) | 1.95 (1.33, 2.85) |
| **WC per 1-SD** | 2.66 (1.94, 3.63) | 2.54 (1.59, 4.06) | 2.43 (1.57, 3.78) | 2.58 (1.63, 4.08) |
| **%BF per 1-SD** | 2.08 (1.53, 2.82) | 2.00 (1.33, 2.99) | 1.90 (1.27, 2.85) | 2.22 (1.45, 3.40) |

Abbreviations: ALT, alanine aminotransferase; AST, aspartate aminotransferase; GGT, gamma-glutamyl transferase.

^a^ Model was stratified by age-at-risk, sex, region, and HBsAg, and adjusted for age at baseline, education, smoking, alcohol, total physical activity, and case-control status in the nested case-control study. Liver enzymes were missing or below the limits of detection in 211 participants.

^b^ Exclusion 1: baseline cancer, cirrhosis, hepatitis, HBsAg positive, heavy alcohol drinkers (men ≥60 g/d and ≥women 40 g/d), and ALT in the top quintile (men ≥31 IU/l and ≥women 24.9 IU/l).

^c^ Exclusion 2: baseline cancer, cirrhosis, hepatitis, HBsAg positive, heavy alcohol drinkers (men ≥60 g/d and ≥women 40 g/d), and AST in the top quintile (men ≥34.6 IU/l and ≥women 31 IU/l).

^d^ Exclusion 3: baseline cancer, cirrhosis, hepatitis, HBsAg positive, heavy alcohol drinkers (men ≥60 g/d and ≥women 40 g/d), and GGT in the top quintile (men ≥43.4 IU/l and ≥women 26.7 IU/l).

# Supplementary Table S7. Adjusted HRs for NAFLD by adiposity when censoring cases with comorbidities prior to NAFLD diagnosis^a^

| **Comorbidities** | **No. of cases** | **BMI** | **WC** | | | **%BF** |
| --- | --- | --- | --- | --- | --- | --- |
|  |  | **HR (95% CI) per 1-SD** | | **HR (95% CI) per 1-SD** | **HR (95% CI) per 1-SD** | |
| **Overall** | 1298 | 2.12 (2.01, 2.22) | | 2.60 (2.45, 2.77) | 2.28 (2.15, 2.41) | |
| **Cardio-metabolic^b^** | 842 | 2.08 (1.96, 2.21) | | 2.53 (2.34, 2.73) | 2.23 (2.07, 2.40) | |
| **Cardio-metabolic and others^c^** | 726 | 2.09 (1.96, 2.23) | | 2.51 (2.31, 2.72) | 2.24 (2.07, 2.42) | |

^a^ Model was stratified by age-at-risk, sex, region, and HBsAg, and adjusted for age at baseline, education, smoking, alcohol, and total physical activity. SD was 3.38 kg/m^2^ for BMI at baseline, 9.74 cm for WC at baseline, and 8.4% for %BF.

^b^ Comorbidities included cardiovascular diseases (I00-I09, I16-I88, I95-I99, I10-I15 [only where fatal]), and diabetes (E10-E14).

^c^ Comorbidities included cardiovascular diseases (I00-I09, I16-I88, I95-I99, I10-I15 [only where fatal]), diabetes (E10-E14), respiratory diseases (J00-J99), and cancer (C00-C97).

# Supplementary Figure S1. Adjusted HRs for NAFLD associated with 1-SD increase in usual level of different measures of adiposity^a^


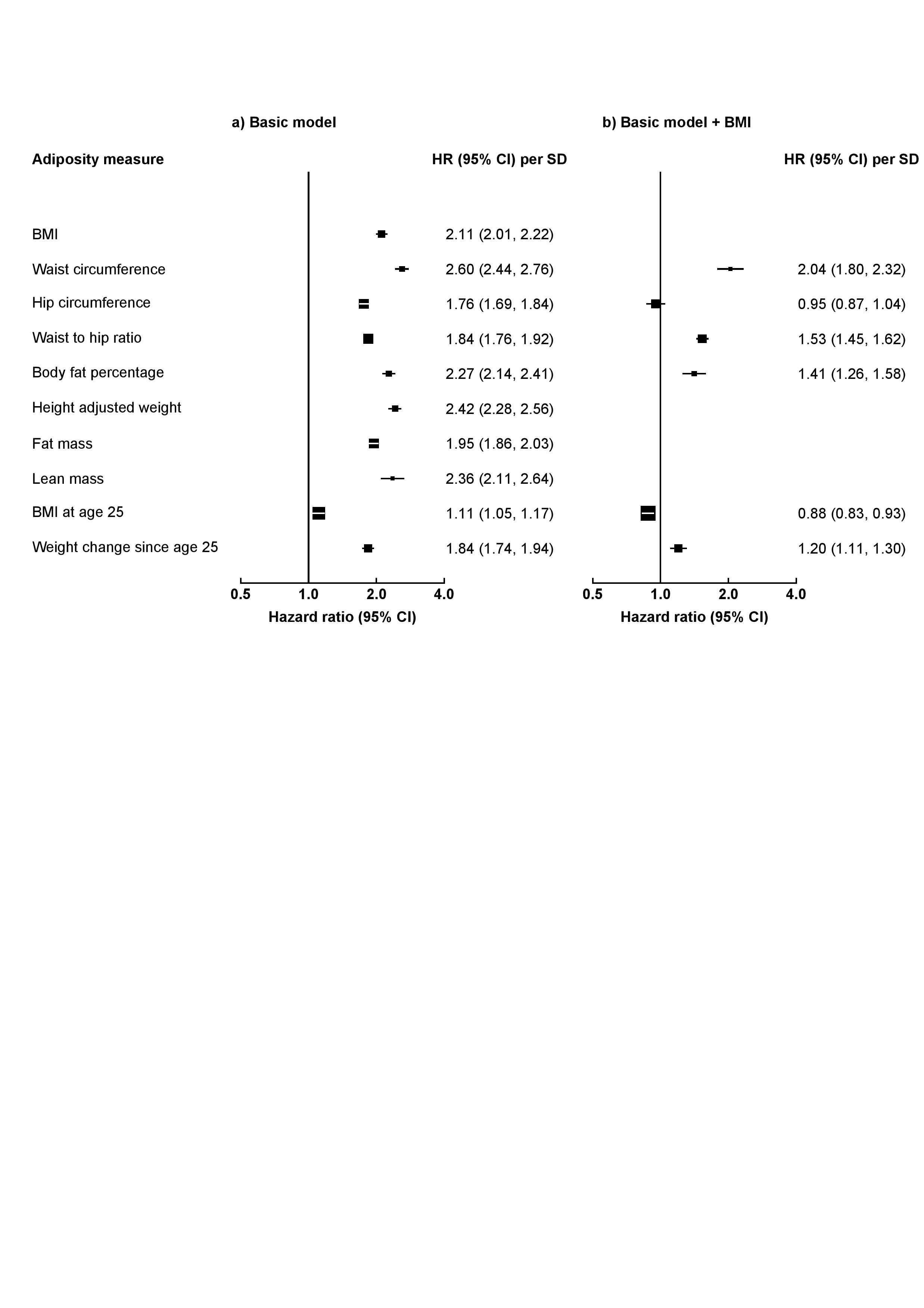


^a^ All adiposity traits were usual levels, except for BMI at age 25 and weight change since age 25. BMI at age 25 was missing in 81,409 participants. Lean mass = (1 - BF%) x weight. Fat mass = BF% x weight.

# Supplementary Figure S2. Adjusted HRs for NAFLD associated with 1-SD increase in usual levels of a) BMI and b) WC in selected subgroups of study population^a^


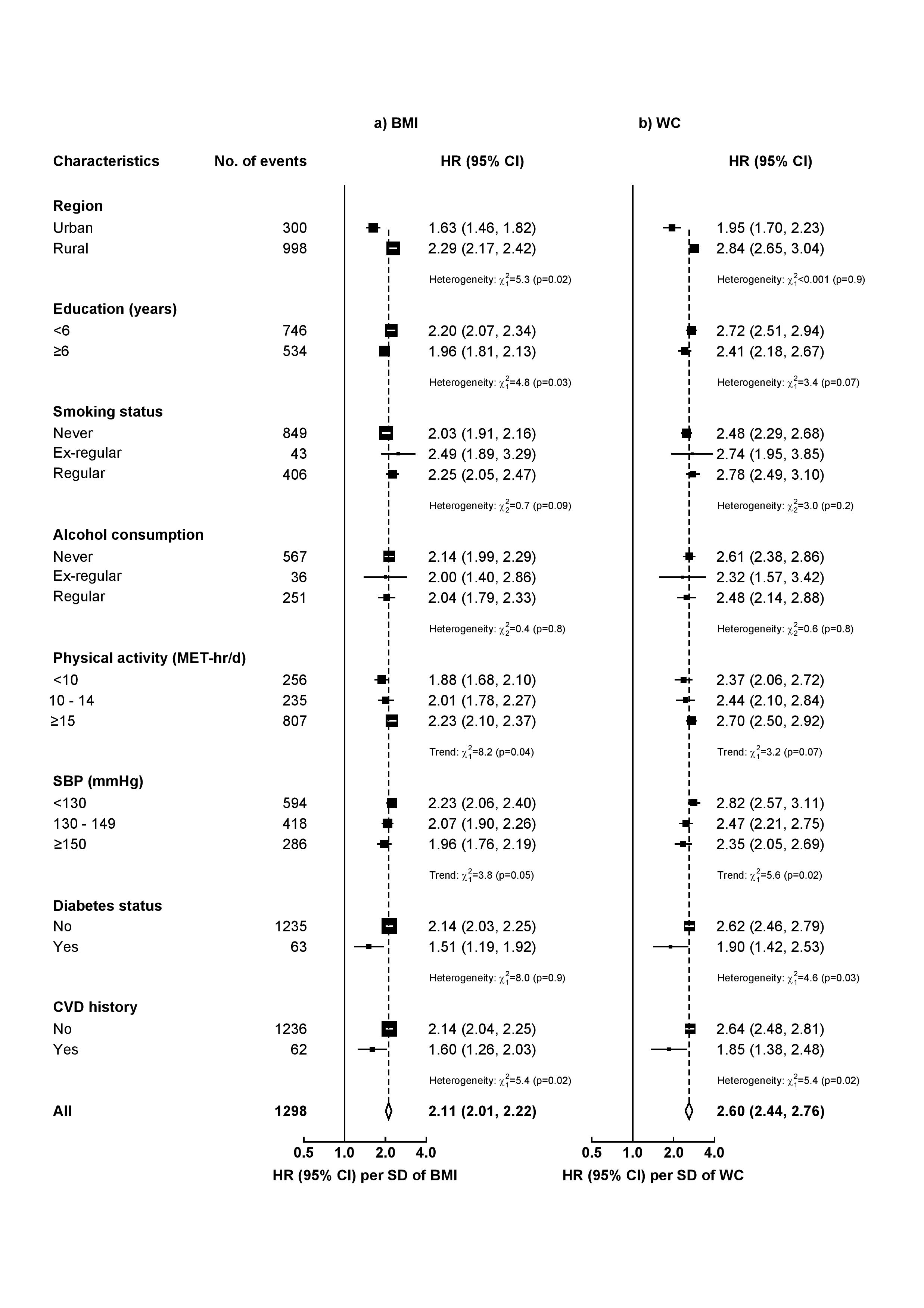


^a^ The analyses were stratified by age-at-risk, sex and study area, and adjusted for education, smoking, and alcohol, where appropriate. SD was 3.38 kg/m^2^ for BMI and 9.74 cm for WC. HR per 1-SD was corrected for regression dilution (regression dilution ratio: BMI=0.93, WC=0.84). Boxes represent subgroup-specific estimates and diamonds represent the overall HR. The sizes of the boxes are proportional to the inverse of the variance of the log hazard ratios.
